# PD-1/PD-L1 Axis, Rather Than High-Mobility Group Alarmins or CD8+ Tumor-Infiltrating Lymphocytes, Is Associated With Survival in Head and Neck Squamous Cell Carcinoma Patients Who Received Surgical Resection

**DOI:** 10.3389/fonc.2018.00604

**Published:** 2018-12-11

**Authors:** Fan Yang, Ziqing Zeng, Jing Li, Yu Zheng, Feng Wei, Xiubao Ren

**Affiliations:** ^1^Department of Immunology, Tianjin Medical University Cancer Institute and Hospital, Tianjin, China; ^2^Department of Biotherapy, Tianjin Medical University Cancer Institute and Hospital, Tianjin, China; ^3^National Clinical Research Center for Cancer, Tianjin, China; ^4^Tianjin Key Laboratory of Cancer Immunology and Biotherapy, Tianjin, China

**Keywords:** HNSCC, HMG alarmin, TILs, Tregs, PD-1/PD-L1 axis

## Abstract

In current studies, the influence of tumor immune microenvironment on tumorigenesis and tumor progression has been widely explored. In the present study, we investigated the expression and significance of high mobility group box 1 (HMGB1), HMG nucleosome-binding protein 1 (HMGN1), the receptor programmed cell death 1 (PD-1) and its ligand programmed cell death ligand 1 (PD-L1) in head and neck squamous cell carcinoma (HNSCC). We explored whether HMGB1 and HMGN1 take part in recruiting T cells to HNSCC microenvironment. Furthermore, we assessed the prognostic value of HMG proteins, TILs, and PD-1/PD-L1 in postoperative patients. Tumor tissue sections were collected from 81 cases of patients with resectable HNSCC. All patients' information was integrated with clinical and pathological records, as well as follow-up data. We used immunohistochemistry to examine the subcellular localization and expression levels of HMGB1 and HMGN1, as well as tumor CD3+, CD8+, FOXP3+ lymphocyte infiltration, and the expression of immune inhibiting molecules PD-1/PD-L1. Results showed that there was no significant difference in the number of CD8+ and FOXP3+ T cells between the two groups with or without HMGB1 cytoplasmic expression in tumor tissues. The number of CD3+ T cells in HMGB1 cytoplasmic expression group (339.39 ± 230.76) was more than that in group without HMGB1 cytoplasmic expression (233.30 ± 230.91, *P* < 0.05). The number of CD3+, CD8+, and FOXP3+ T cells in HMGN1 cytoplasmic expression group [400.74 ± 224.04, 158.10 ± 112.10, 36.00(15.00, 69.00)] was more than that in group without HMGN1-cytoplasmic expression [222.84 ± 217.78, *P* < 0.01; 105.10 ± 108.25, *P* < 0.05; 13.00(6.75, 32.25), *P* < 0.01]. The positive rates of PD-1 and PD-L1 in tumor tissues were 29.6 and 67.9%, respectively. Multivariate analysis suggested that tumor expression of PD-L1 was an independent prognostic factor and PD-L1 overexpression indicated a poor overall survival (OS) and disease-free survival (DFS). Taken together, we concluded that HMGB1 and HMGN1 secreted by cancer cells may relate to recruitment of tumor infiltrating lymphocytes (TILs) in HNSCC. PD-1/PD-L1 axis, rather than HMG proteins or CD8+ tumor-infiltrating lymphocytes, has a critical role in tumor immune microenvironment and could predict the outcome of HNSCC patients who received surgical resection.

## Introduction

Head and neck squamous cell carcinoma (HNSCC) is the most frequent malignancy in the head and neck region, with about 650,000 new cases reported annually worldwide. The epidemiology of HNSCC has changed dramatically in recent decades (1). The incidence of oral cavity and laryngeal squamous cell cancers has declined, while the incidence of oropharyngeal squamous cell carcinoma (OPSCC) has increased. Chronic infection of high-risk human papillomavirus (HPV) has been considered as one of critical factors of most HNSCCs. The prevalence of HPV in HNSCC is 60% (2), and rate is steadily increasing. In general, HNSCC is divided into two subclasses: HPV positive (HPV+) and HPV negative (HPV–). In addition, studies have demonstrated p16 was a reliable surrogate for HPV tumor status only in the OP but not in non-OP HNSCCs (3). Despite the rapid development of diagnostic and therapeutic approaches in recent years, the 5-years survival rate of patients suffering HNSCC is still < 50%, which remain unchanged over the past three decades. Due to intensive research on cancer immunology and tumor molecular biology, cancer immunotherapy has shown great promise for the HNSCC therapy development.

As a complex disease, cancer involves interactions between the tumor and the immune system. Immune cells in the tumor microenvironment play an important role in tumor-associated immune responses, which may regulate tumor progression and determine the prognosis. However, immune cells infiltrating tumors are highly heterogeneous. Most of the components of myeloid and lymphoid compartments are major representatives, such as Th1 and cytotoxic T lymphocytes (CTLs), regulatory T (Treg) cells, B cells, macrophages. Among all immune cells, T cell immune infiltration is the most significant predictor of patient survival. CD3 is a common differentiation antigen of mature T lymphocytes, expressed on the surface of mature T cells. CD8 + cytotoxic T cells are the main antitumor effector cells that kill tumor cells by releasing perforin or promoting apoptosis. Forkfead box P3 (FOXP3)+ Treg cells play the role of inhibiting anti-tumor immune response. Thus, in tumor-associated immune responses, the function of TILs is a combined effect regulated by interactions between different subsets. The prognostic and predictive significance of tumor-infiltrating lymphocytes (TILs) has been established for a range of tumors (4, 5).

High-mobility group (HMG) proteins are the most abundant non-histone chromosomal proteins that bind nucleosomes and regulate chromosome architecture and gene transcription. HMG proteins can be classified into HMGA, HMGB, and HMGN families according to unique structural motifs. On the other hand, HMG proteins in the extracellular milieu, which are released from injured, dying or stressed cells, have also been recognized as danger-associated molecular pattern (DAMP) molecules based on their shared chemical property of hydrophobicity and function of stimulating the immune system (6). As “alarmins,” HMGB1 and HMGN1 are shown to exhibit similar chemoattracting and activating effects on dendritic cells (DCs), as well as the capacity to enhance innate and adaptive immune responses. A study found that high cytoplasmic expression of HMG proteins correlated with high levels of TILs in HER2-positive breast cancers (7). However, the expression patterns of HMG proteins in HNSCC tissues are largely unknown.

Although some studies confirm the association between TILs and survival in HNSCC patients, the prognostic role of TILs is still controversial. One reason might be that PD-1/PD-L1 axis plays a role in silencing immune surveillance by rendering CD8+ cytotoxic T cells anergic. In the present study, we assessed the subcellular localization and levels of HMGB1 and HMGN1 expression. We also further evaluated the association of HMG proteins and TILs in HNSCC tissues and explored their correlation with clinicopathological variables, including patient survival. In addition, we also analyzed the predicting value of PD-1/PD-L1 status on postoperative outcome.

## Materials and Methods

### Patients and Tissue Specimens

A total of 81 HNSCC patients who underwent surgery from Department of Head and Neck Oncology, Tianjin Medical University Cancer Institute and Hospital (TMUCIH), Tianjin, China, between August 2009 and October 2013 were enrolled. All patients had formalin-fixed, paraffin-embedded tissue samples available for analysis and were diagnosed as HNSCC pathologically for the first time, within the enrollment time. The TNM staging system was used to classify the tumors in accordance with the American Joint Committee on Cancer classification. The Institutional Review Board approved the protocol for this study, and written informed consent was obtained from each patient before enrollment.

All patients were followed up until December 2017. The definition of overall survival (OS) was the period from the surgery to death of any reason or to the time of last follow-up. disease-free survival (DFS) was defined as the period after curative treatment (surgery) to the time when recurrence was diagnosed or to the last date of follow-up when no disease can be detected. Patients lost to follow-up were censored as of the last day of follow-up. Patients who died from causes other than HNSCC were censored at the date of death.

### Immunohistochemistry (IHC)

Primary antibodies used for IHC of head and neck squamous cell carcinoma tissues included anti-HMGB1 (1:500, Abcam, USA), anti-HMGN1 antibody (1:300, Cell Signaling Technology, USA), anti-CD3 antibody (1:100, Abcam, USA), anti-CD8 antibody (1:100, Abcam, USA), anti-FOXP3 antibody (1:100, Abcam, USA), anti-PD-1 antibody (1:200, Proteintech, USA), or anti-PD-L1 antibody (1:400, Proteintech, USA). In brief, paraffin-embedded specimens were baked for 1 h at 70°C, following by de-paraffinating, then rehydrated with xylene and ethanol. The sections were pretreated with antigen retrieval buffers (citrate buffer or ethylene diamine tetraacetic acid) in a microwave for 15 min and restored to room temperature spontaneously. Specimens were soaked in 0.3% hydrogen peroxide for 20 min to inhibit the endogenous peroxidase activity. Then we incubated the tissues with appropriately diluted primary antibodies at 4°C overnight. After washing with PBS, added a secondary antibody of undiluted horseradish peroxidase (HRP)-conjugated goat anti-rabbit or goat anti-mouse IgG polyclonal antibody (Maixin, China) to the samples at 37°C for 30 min. After rinsing, samples were incubated with 3,3'-diaminobenzidine (DAB) chromogen substrate (ZSGB-BIO, China). Then the slides were soaked in chromogen substrate to stain the nucleus. At last, the tissue slides were covered with a glass coverslip then evaluated by light microscope.

Tumor sections were accessed by a consensus of two pathologists who did not know the characteristics of the tissue. Five images were randomly taken at high magnification on each slide. Positive HMGB1 and HMGN1 expression existed in nucleus of tumor cells, mesenchymal cells, as well as immune cells. Based on the staining intensity of nucleus in tumor cells, tumor tissues were divided into high expression group and low expression group. According to whether HMGB1 or HMGN1 expressed in the cytoplasm of tumor cells, the tissue sections were divided into cytoplasmic positive group and cytoplasmic negative group. In the case of CD3+, CD8+, and FOXP3+ staining, the positive cells per core were counted manually and average counts of five fields were used for analysis. Positive CD3+, CD8+, or FOXP3+ expression represent the number of total T cells, CTLs or Tregs in the tumor microenvironment, respectively.

In the 81 cases of HNSCC, tissue sections were considered as PD-1 positive when at least 10% of lymphocytes in tumor microenvironment showed PD-1 staining. PD-L1 positive were defined as tumor cells having membranous or cytoplasmic staining. According to previous studies, < 5% tumor cells staining were defined as negative (–, scored as 0). For samples with PD-L1 positive ≥5%, based on the staining intensity from light brown to general brown and dark brown, further divided the expression levels into three degrees: weakly (+, recorded as 1), moderately (++, recorded as 2), and strongly positive (+++, recorded as 3) correspondingly. Negative and weak positive expression was considered as low expression while moderately and strongly positive expression was described as high expression.

### Statistical Analysis

All statistical analyses were performed using Statistical Package for the Social Sciences version 17.0 (IBM Corp., USA), and the normality of the data was evaluated using the Kolmogorov-Smirnov test. Given that there was no clinically defined cutoff points for the number of TILs in tumor microenvironment, the median numbers of CD3+, CD8+, and FOXP3+ T cells (227 for CD3+, 90 for CD8+, and 18 for FOXP3+) were used as a cutoff point to define the TILs-low and TILs-high groups. Person chi-square (χ2) test or Fisher's exact test were used to compare categorical variables. The correlations between HMGB1 or HMGN1 expression and TILs were assessed using Spearman's correlation analysis. Kaplan-Meier analysis was the method used to calculate the overall survival (OS) and disease-free survival (DFS), then the OS and DFS was compared by log-rank test. Multivariable analysis of the independent factors which were correlated with survival was performed by the Cox proportional hazard model. Statistically significant is considered as the two-tailed *P* < 0.05.

## Results

### Patient Characteristics and Baseline Clinicopathologic Characteristics

This analysis included 81 HNSCCs, including cancers of the oropharynx (*n* = 17), oral cavity/lip (*n* = 38), larynx (*n* = 17), and hypopharynx (*n* = 9). Patients were predominantly male (80.2%) and the majority of tumor tissue samples were moderately (58.0%) differentiated. Median age was 56 years (interquartile range, 49–64 years), and most patients had a history of ever tobacco and alcohol use.

### Expression of HMG Proteins in Patients With HNSCC and its Relationship With TILs

**Figure 1:**
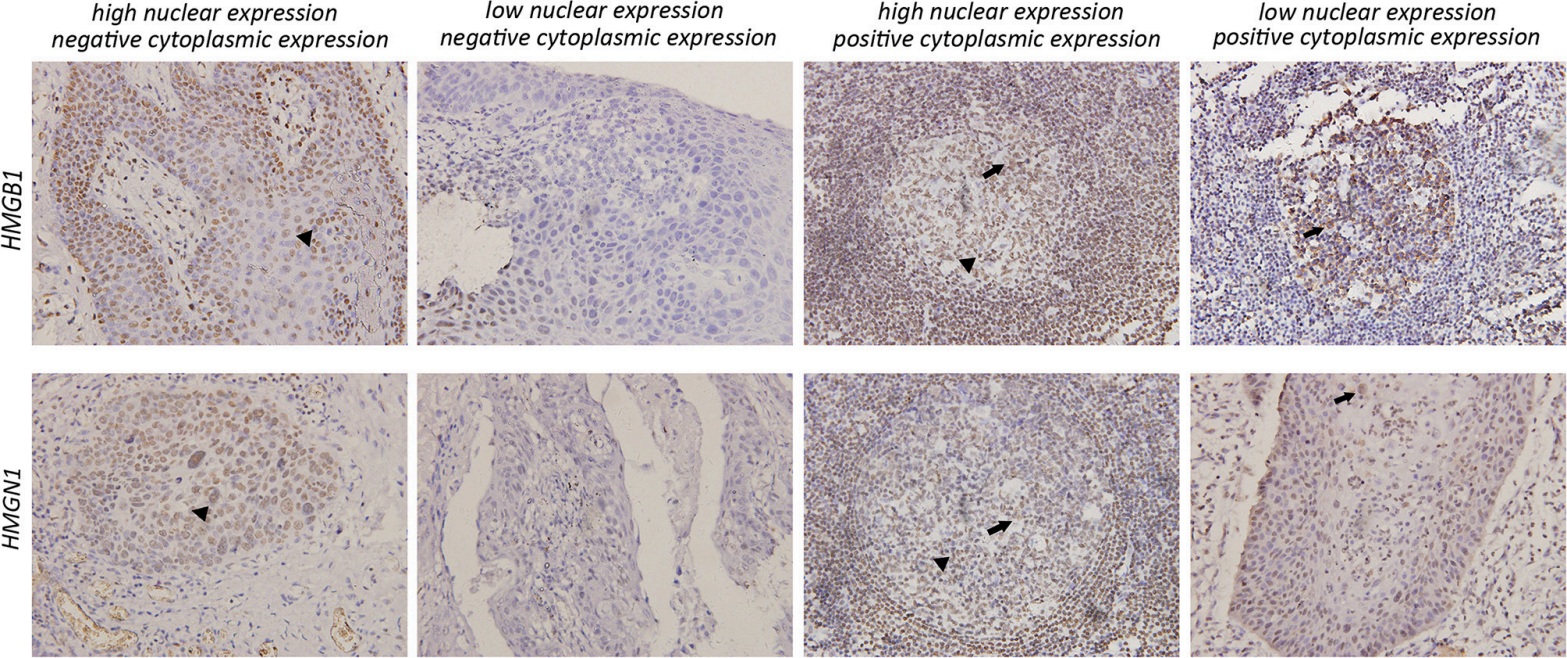
Various expression patterns of HMGB1 and HMGN1 in tumor cells (×200). In tumors characterized by positive cytoplasmic expression of HMGB1 and HMGN1, a large number of infiltrating lymphocytes could be observed around tumor cell nest. All images show both nuclear expression (*filled arrowhead*) and cytoplasmic expression (*arrowhead*) of tumor cells.

Epithelial cells and lymphocytes showed nuclear expression of HMGB1 and HMGN1. However, various immunostaining patterns of these two proteins could be observed in carcinoma cells (Figure 1). The correlation of HMG alarmins expression with clinicopathological characteristics were shown in Tables 1 and 2. Positive cytoplasmic expression of HMGB1 was significantly associated with a low lymph node positive rate (*P* = 0.004) and p16 negativity (*P* = 0.049). Besides, nuclear expression of HMGB1 was correlated with drinking status (*P* = 0.040), while nuclear expression of HMGN1 was associated with p16 status (*P* = 0.033). The nuclear expression of HMGB1 and HMGN1, and the cytoplasmic expression of both proteins were significantly correlated with each other (rho = 0.456, *P* < 0.001 for nuclear expression; rho = 0.518, *P* < 0.001 for cytoplasmic expression).

**Table 1:** Comparison of pathological variables based on the cytoplasmic expression of HMG proteins in patients with HNSCC.

**Parameter**	**Overall**	**Cytoplasmic HMGB1**			**Cytoplasmic HMGN1**		
		**Negative (%)**	**Positive (%)**	***P*-value**	**Negative (%)**	**Positive (%)**	***P*-value**
Patient number	81 (100.0)	37 (45.7)	44 (54.3)		50 (61.7)	31 (38.3)	
Gender				0.863			0.943
Male	65 (80.2)	30 (37.0)	35 (43.2)		40 (49.4)	25 (30.9)	
Female	16 (19.8)	7 (8.7)	9 (11.1)		10 (12.3)	6 (7.4)	
Age (years)				0.461			0.079
< 60	49 (60.5)	24 (29.6)	25 (30.9)		34 (42.0)	15 (18.5)	
≥60	32 (39.5)	13 (16.1)	19 (23.4)		16 (19.7)	16 (19.8)	
Localization				0.130			0.776
Oropharynx	17 (21.0)	5 (6.2)	12 (14.8)		11 (13.6)	6 (7.4)	
Non-oropharynx	64 (79.0)	32 (39.5)	32 (39.5)		39 (48.1)	25 (30.9)	
Histology				0.155			0.160
Well differentiated	18 (22.2)	9 (11.1)	9 (11.1)		13 (16.0)	5 (6.2)	
Moderately differentiated	47 (58.0)	21 (25.9)	26 (32.1)		29 (35.8)	18 (22.2)	
Poorly differentiated	16 (19.8)	7 (8.7)	9 (11.1)		8 (9.9)	8 (9.9)	
T classification				0.626			0.221
T1	23 (28.4)	9 11.1)	14 (17.3)		16 (19.8)	7 (8.6)	
T2	25 (30.9)	13 (16.0)	12 (14.8)		13 (16.0)	12 (14.8)	
T3	10 (12.3)	5 (6.2)	5 (6.2)		7 (8.6)	3 (3.7)	
T4	23 (28.4)	10 (12.3)	13 (16.0)		14 (17.3)	9 (11.1)	
Lymph node metastasis				0.004			0.198
Negative (N0)	49 (60.5)	16 (19.8)	33 (40.7)		33 (40.7)	16 (19.8)	
Positive (N1–3)	32 (39.5)	21 (25.9)	11 (13.6)		17 (21.0)	15 (18.5)	
TNM stage				0.594			0.381
I	20 (24.7)	9 (11.1)	11 (13.6)		13 (16.0)	7 (8.6)	
II	13 (16.0)	8 (9.9)	5 (6.2)		7 (8.6)	6 (7.4)	
III	11 (13.6)	4 (4.9)	7 (8.7)		8 (9.9)	3 (3.7)	
IV	37 (45.7)	16 (19.8)	21 (25.9)		22 (27.2)	15 (18.5)	
Tobacco smoking				0.296			0.365
Non-smoker	29 (35.8)	11 (13.6)	18 (22.2)		16 (19.8)	13 (16.0)	
Smoker	52 (64.2)	26 (32.1)	26 (32.1)		34 (42.0)	18 (22.2)	
Alcohol consumption				0.843			0.505
Non-drinker	38 (46.9)	15 (18.5)	13 (16.0)		22 (27.2)	16 (19.8)	
Drinker	43 (53.1)	22 (27.2)	21 (25.9)		28 (34.6)	15 (18.5)	
p16 status				0.049			0.642
Negative	26 (32.1)	16 (19.8)	10 (12.3)		17 (21.0)	9 (11.1)	
Positive	55 (67.9)	21 (25.9)	34 (42.0)		33 (40.7)	22 (27.2)	

**Table 2:** Comparison of pathological variables based on the nuclear expression of HMG proteins in patients with HNSCC.

**Parameter**	**Overall**	**Nuclear HMGB1**			**Nuclear HMGN1**		
		**Low (%)**	**High (%)**	***P*-value**	**Low (%)**	**High (%)**	***P*-value**
Patient number	81 (100.0)	35 (43.2)	46 (56.8)		39 (48.1)	42 (51.9)	
Gender				0.540			0.869
Male	65 (80.2)	27 (33.3)	38 (46.9)		31 (38.3)	34 (42.0)	
Female	16 (19.8)	8 (9.9)	8 (9.9)		8 (9.9)	8 (9.9)	
Age (years)				0.402			0.273
< 60	49 (60.5)	23 (28.4)	26 (32.1)		26 (32.1)	23 (28.4)	
≥60	32 (39.5)	12 (14.8)	20 (28.9)		13 (16.0)	19 (23.5)	
Localization				0.144			0.656
Oropharynx	17 (21.0)	10 (12.3)	7 (8.6)		9 (16.0)	8 (9.9)	
Non-oropharynx	64 (79.0)	25 (30.9)	39 (48.1)		30 (37.0)	34 (42.0)	
Histology				0.229			0.147
Well differentiated	18 (22.2)	13 (16.0)	5 (6.2)		11 (13.6)	7 (8.6)	
Moderately differentiated	47 (58.0)	16 (19.8)	31 (38.3)		23 (28.4)	24 (29.6)	
Poorly differentiated	16 (19.8)	6 (7.4)	10 (12.3)		5 (6.2)	11 (13.6)	
T classification				0.211			0.687
T1	23 (28.4)	13 (16.0)	10 (12.3)		10 (12.3)	13 (16.0)	
T2	25 (30.9)	10 (12.3)	15 (18.5)		13 (16.0)	12 (14.8)	
T3	10 (12.3)	4 (4.9)	6 (7.4)		5 (6.2)	5 (6.2)	
T4	23 (28.4)	8 (9.9)	15 (18.5)		11 (13.6)	12 (14.8)	
Lymph node metastasis				0.402			0.853
Negative (N0)	49 (60.5)	23 (28.4)	26 (32.1)		24 (29.6)	25 (30.9)	
Positive (N1–3)	32 (39.5)	12 (14.8)	20 (28.9)		15 (18.5)	17 (21.0)	
TNM stage				0.096			0.973
I	20 (24.7)	12 (14.8)	8 (9.9)		9 (16.0)	11 (13.6)	
II	13 (16.0)	6 (7.4)	7 (8.6)		8 (9.9)	5 (6.2)	
III	11 (13.6)	5 (6.2)	6 (7.4)		6 (7.4)	5 (6.2)	
IV	37 (45.7)	12 (14.8)	25 (30.9)		16 (19.8)	21 (25.9)	
Tobacco smoking				0.492			0.986
Non-smoker	29 (35.8)	14 (17.3)	15 (18.5)		14 (19.8)	15 (18.5)	
Smoker	52 (64.2)	21 (25.9)	31 (38.3)		25 (42.0)	27 (33.3)	
Alcohol consumption				0.040			0.895
Non-drinker	38 (46.9)	21 (25.9)	17 (21.0)		18 (22.2)	20 (24.7)	
Drinker	43 (53.1)	14 (17.3)	29 (35.8)		21 (25.9)	22 (27.2)	
p16 status				0.396			0.033
Negative	26 (32.1)	13 (16.0)	13 (16.0)		17 (21.0)	9 (11.1)	
Positive	55 (67.9)	22 (27.2)	33 (40.7)		22 (27.2)	33 (40.7)	

The correlation between cancer tissue expressed HMGB1/HMGN1 and stromal immune cell infiltrate were then observed. The correlation of TILs with clinicopathological characteristics were shown in Table 3. High CD8+ T cell count was significantly associated with drinking. As shown in Table 4, nuclear expression of HMGB1 was inversely correlated with CD3+ T cells, CD8+ CTLs, and FOXP3+ Tregs, while cytoplasmic expression of HMGN1 was significantly associated with these three groups of TILs. Further analysis showed there was no significant difference in the number of CD8+ and FOXP3+ regulatory T cells between the two groups with or without HMGB1 cytoplasmic expression in tumor tissues [142.77 ± 118.29 vs. 104.70 ± 101.94, and 20(9.00, 53.75) vs. 18.00(6.00, 43.00)]. The number of CD3+ T cells in HMGB1 cytoplasmic expression group (339.39 ± 230.76) was more than that in group without HMGB1 cytoplasmic expression (233.30 ± 230.91, *P* < 0.05), suggesting that cytoplasmic expression of HMGB1 in cancer cells is associated with total T lymphocytes infiltrated in the tumor microenvironment. The CD3+ T cell count, CD8+ T cell count and FOXP3+ regulatory T cell count in HMGN1 cytoplasmic expression group [400.74 ± 224.04, 158.10 ± 112.10, 36.00(15.00, 69.00)] were more than that in group without HMGN1-cytoplasmic expression [222.84 ± 217.78, *P* < 0.01; 105.10 ± 108.25, *P* < 0.05; 13.00(6.75, 32.25), *P* < 0.01], suggesting that cytoplasmic expression of HMGN1 in cancer cells is associated with CD3+ T lymphocytes, CD8+ CTLs and FOXP3+ regulatory T cells infiltrated in the tumor microenvironment.

**Table 3:** Comparison of pathological variables based on the TILs in tumor microenvironment.

**Parameter**	**Overall**	**CD3+** **total T cells**			**CD8+** **CTLs**			**FOXP3+** **Tregs**		
		**Low (%)**	**High (%)**	***P* value**	**Low (%)**	**High (%)**	***P* value**	**Low (%)**	**High (%)**	***P* value**
Patient number	81 (100.0)	41 (50.6)	40 (49.4)		41 (50.6)	40 (49.4)		41 (50.6)	40 (49.4)	
Gender				0.956			0.956			0.289
Male	65 (80.2)	33 (40.7)	32 (39.5)		33 (40.7)	32 (39.5)		31 (38.3)	34 (42.0)	
Female	16 (19.8)	8 (9.9)	8 (9.9)		8 (9.9)	8 (9.9)		10 (12.3)	6 (7.4)	
Age (years)				0.928			0.715			0.928
< 60	49 (60.5)	25 (30.9)	24 (29.6)		24 (29.6)	25 (30.9)		25 (30.9)	24 (29.6)	
≥60	32 (39.5)	16 (19.8)	16 (19.8)		17 (21.0)	15 (18.5)		16 (19.8)	16 (19.8)	
Localization				0.741			0.829			0.446
Oropharynx	17 (21.0)	8 (9.9)	9 (11.1)		9 (11.1)	8 (9.9)		10 (12.3)	7 (8.6)	
Non-oropharynx	64 (79.0)	33 (40.7)	31 (38.3)		32 (39.5)	32 (39.5)		31 (38.3)	33 (40.7)	
Histology				0.150			0.150			0.150
Well differentiated	18 (22.2)	6 (7.4)	12 (14.8)		6 (7.4)	12 (14.8)		9 (11.1)	9 (11.1)	
Moderately differentiated	47 (58.0)	25 (30.9)	22 (27.2)		25 (30.9)	22 (27.2)		25 (30.9)	22 (27.2)	
Poorly differentiated	16 (19.8)	10 (12.3)	6 (7.4)		10 (12.3)	6 (7.4)		7 (8.6)	9 (11.1)	
Lymph node metastasis				0.928			0.715			0.586
Negative (N0)	49 (60.5)	25 (30.9)	24 (29.6)		24 (29.6)	25 (30.9)		26 (32.1)	23 (28.4)	
Positive (N1–3)	32 (39.5)	16 (19.8)	16 (19.8)		17 (21.0)	15 (18.5)		15 (18.5)	17 (21.0)	
TNM stage				0.441			0.221			0.893
I/II	33 (40.7)	15 (18.5)	18 (22.2)		14 (17.3)	19 (23.5)		17 (21.0)	16 (19.8)	
III/IV	48 (59.3)	26 (32.1)	22 (27.2)		27 (33.3)	21 (25.9)		24 (29.6)	24 (29.6)
Tobacco smoking				0.882			0.753			0.753
Non-smoker	29 (35.8)	15 (18.5)	14 (17.3)		14 (17.3)	15 (18.5)		14 (17.3)	15 (18.5)	
Smoker	52 (64.2)	26 (32.1)	26 (32.1)		27 (33.3)	25 (30.9)		27 (33.3)	25 (30.9)	
Alcohol consumption				0.088			0.009			0.420
Non-drinker	34 (42.0)	21 (25.9)	13 (16.0)		23 (28.4)	11 (13.6)		19 (23.5)	15 (18.5)	
Drinker	47 (58.0)	20 (24.7)	27 (33.3)		18 (22.2)	29 (35.8)		22 (27.2)	25 (30.9)	
p16 status				0.132			0.132			0.304
Negative	26 (32.1)	10 (12.3)	16 (19.8)		10 (12.3)	16 (19.8)		11 (13.6)	15 (18.5)	
Positive	55 (67.9)	31 (38.3)	24 (29.6)		31 (38.3)	24 (29.6)		30 (37.0)	25 (30.9)	

**Table 4:** Correlation coefficients between each continuous variable.

**Parameter**	**Cytoplasmic HMGB1**	**Nuclear HMGN1**	**Cytoplasmic HMGN1**	**CD3+ total T cells**	**CD8+ cytotoxic T cells**	**FOXP3+ Tregs**
Nuclear HMGB1	−0.149 (0.183)	0.456 (0.000)	−0.082 (0.465)	−0.316 (0.004)	−0.246 (0.027)	−0.254 (0.022)
Cytoplasmic HMGB1		0.009 (0.935)	0.518 (0.000)	0.253 (0.022)	0.177 (0.115)	0.100 (0.376)
Nuclear HMGN1			−0.004 (0.973)	−0.269 (0.015)	−0.202 (0.071)	−0.141 (0.211)
Cytoplasmic HMGN1				0.403 (0.000)	0.272 (0.014)	0.311 (0.005)
CD3+ total T cells					0.800 (0.000)	0.532 (0.000)
CD8+ cytotoxic T cells						0.310 (0.005)

### HMG Alarmins, Tumor-Infiltrating Lymphocytes, PD-1/PD-L1 Status and Survival

**Figure 2:**
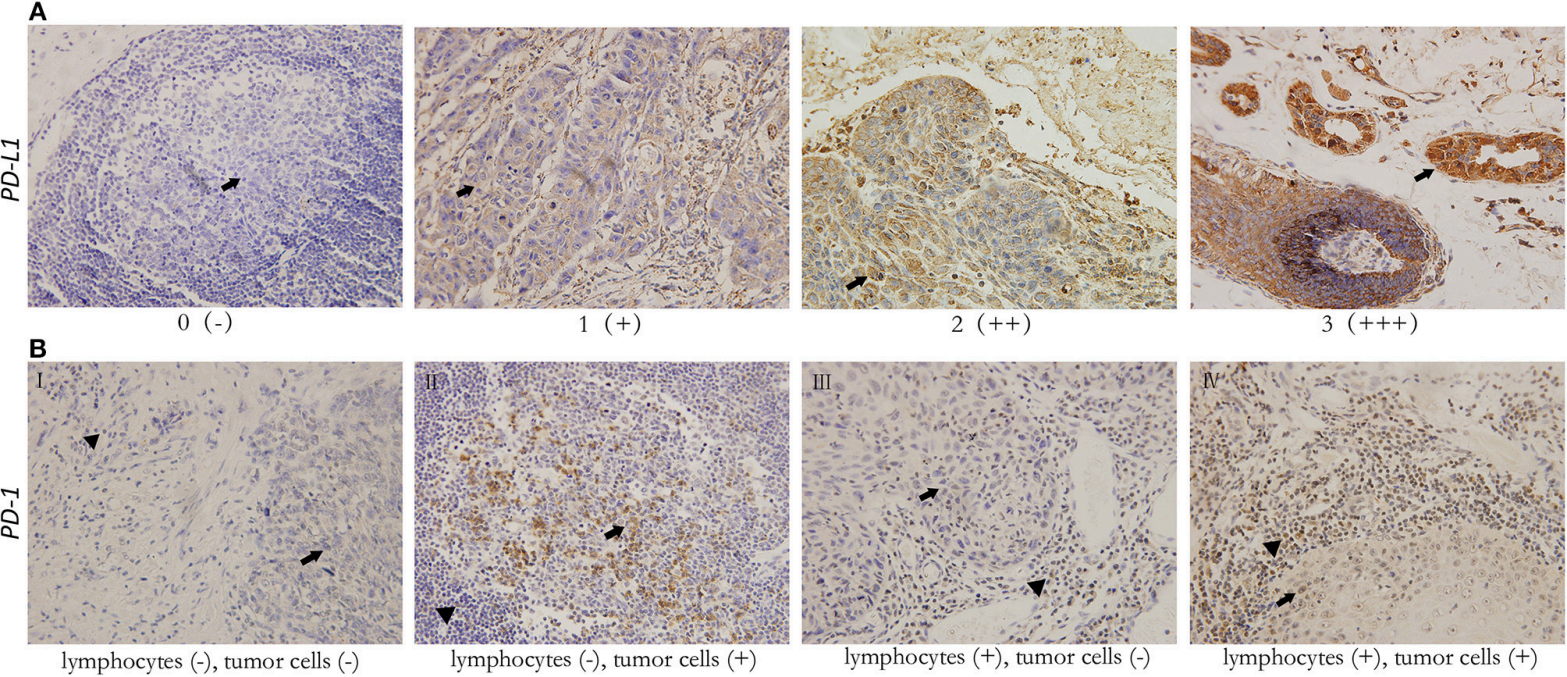
Representative immunohistochemical staining for PD-L1 (×200) in head and neck squamous cell carcinoma, and PD-1 (×200) in both tumor cells and tumor-infiltrating lymphocytes. All images show both tumor cell area (*arrowhead*) and lymphocytes (*filled arrowhead*). **(A)** For PD-L1, scores 0, 1, 2, 3 represent negative (–), weak positive (+), moderate positive (++), and strong positive (+ + +) expression, respectively. **(B)** Various expression patterns of PD-1 in tumor tissues (I. PD-1- tumor with PD-1- TILs II. PD-1+ tumor with PD-1- TILs III. PD-1- tumor with PD-1+ TILs IV. PD-1+ tumor with PD-1+ TILs).

**Figure 3:**
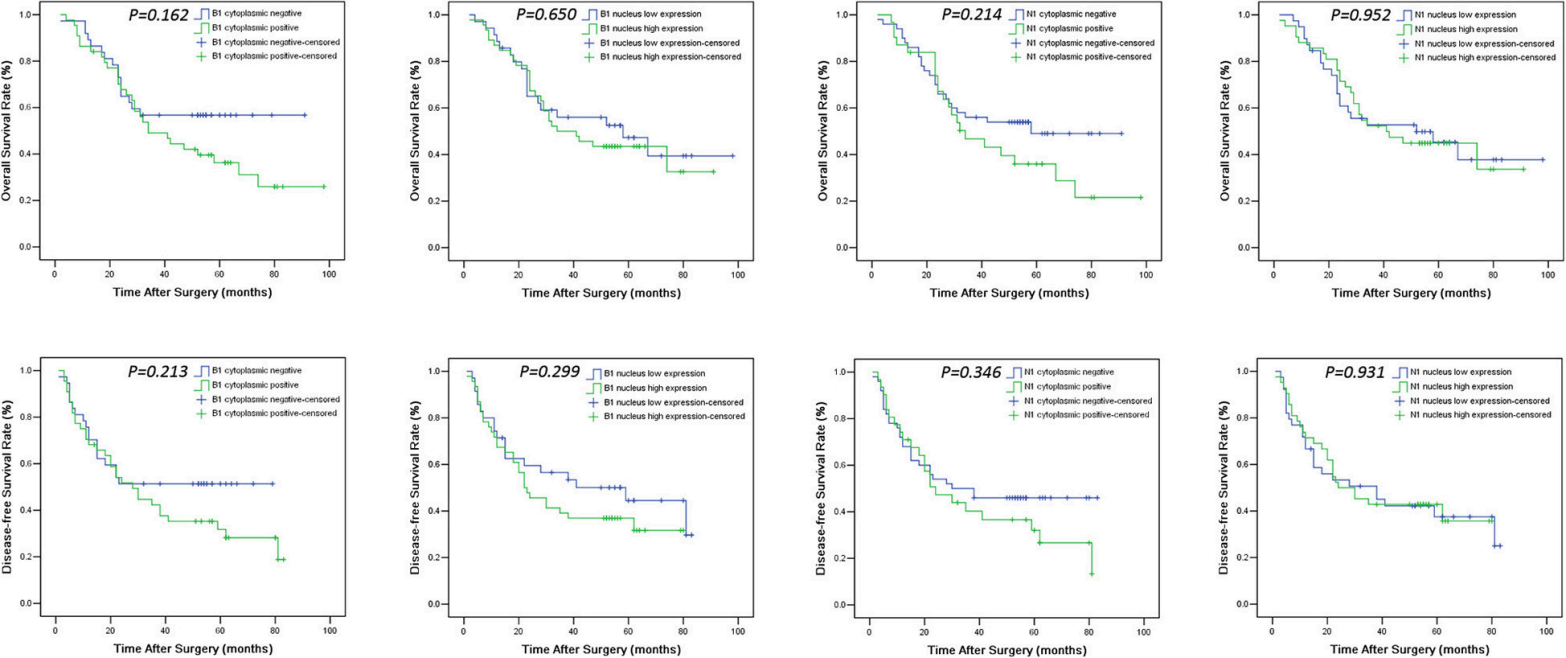
Overall survival (OS) and disease-free survival (DFS) Kaplan-Meier curves of HNSCC patients according to expression levels of HMGB1 and HMGN1 in nucleus and cytoplasm. For determination of the *p*-values the log-rank test was used.

**Figure 4:**
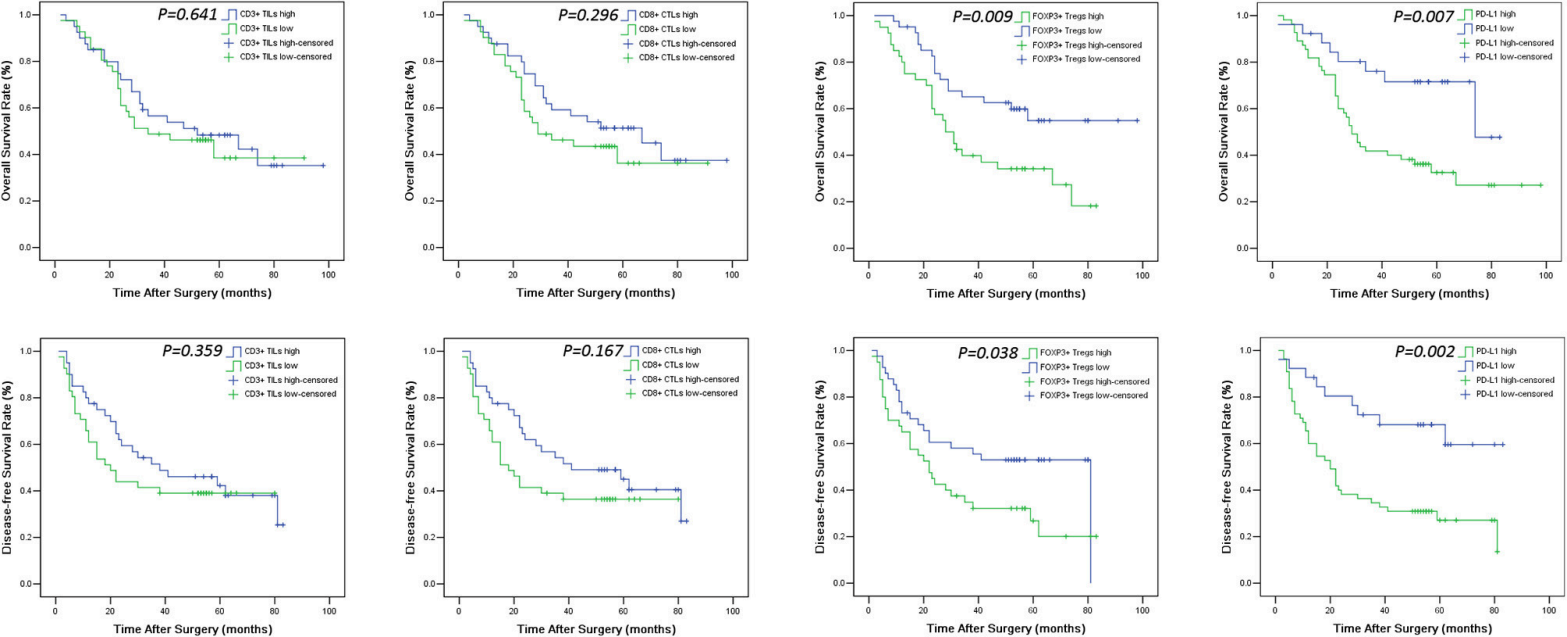
OS and DFS survival Kaplan-Meier curves of CD3+ TILs, CD8+ CTLs, FOXP3+ Tregs count and PD-L1 expression level in the tumor. Log-rank test was used to compare the above curves.

The levels of cancer expressed PD-1 and PD-L1 were also detected by IHC. PD-1 was considered as negative or positive according to the staining proportion, and PD-L1 was recorded as 0, 1, 2, and 3, respectively representing negative, weakly positive, moderately positive and strongly positive, in accord with both the intensity and proportion of staining (Figure 2). The positive rates of PD-1 and PD-L1 in tumor tissues were 29.6 and 67.9%, respectively. Next, we investigated the role of HMG alarmins expression, TILs pattern, and PD-1/PD-L1 status on patients' survival. Optimal cut-offs for low vs. high staining were made as described in the statistical methods. Patients with negative HMGB1 or HMGN1 cytoplasmic expression seemed like to show an improved effect on OS and DFS, even though there was no statistical significance (Figure 3). For TILs, low FOXP3+ regulatory T cell count but not CD3+ T cell count and CD8+ T cell count was suggested to have a favorable influence on OS and DFS (Figure 4).

The median follow-up duration was 41 months (ranging from 2 to 98 months). At the end of the follow-up period, there were 48 deaths and 33 survivals. Patients' 1-, 3-, and 5-years OS rate were 88, 51, and 21%, respectively, and the corresponding DFS rate was 70, 44, and 17%, respectively. Univariate analysis showed that OS has significant correlation with TNM stage (*P* = 0.001), tumor size (*P* = 0.014), lymph node metastasis (*P* = 0.000), Treg count (*P* = 0.009), and PD-L1 expression (*P* = 0.007), while DFS was directly influenced by histology (*P* = 0.007), TNM stage (*P* = 0.001), tumor size (*P* = 0.010), lymph node metastasis (*P* = 0.002), Treg count (*P* = 0.038), and PD-L1 expression (*P* = 0.002). Multivariate analysis showed lymph node metastasis, Treg count and PD-L1 expression on tumor cells were independent prognostic factors for OS, while TNM stage, Treg count and PD-L1 expression could be regarded as independent prognostic factors of DFS (all *P* < 0.05, Tables 5, 6). Patients with low PD-L1 expression on tumor cells had a more favorable outcome.

**Table 5:** Univariate and multivariate analysis of clinicopathological factors in regard to OS in patients with HNSCC.

**Characteristics**	**Cases, n (%)**	**Univariate**		**Multivariate**	
		**Survival time (month)**	***P*-value**	**HR (95% CI)**	***P*-value**
**GENDER**					
Male	65 (80.2)	53.18 ± 4.80	0.496		
Female	16 (19.8)	54.79 ± 7.22			
**AGE (YEARS)**					
< 60	49 (60.5)	54.00 ± 5.45	0.861		
≥60	32 (39.5)	54.22 ± 6.18			
**LOCALIZATION**					
Oropharynx	17 (21.0)	40.98 ± 5.80	0.817		
Non-oropharynx	64 (79.0)	55.07 ± 4.75			
**HISTOLOGY**					
Well-differentiated	18 (22.2)	64.18 ± 7.23	0.051		
Moderately differentiated	47 (58.0)	52.43 ± 5.26			
Poorly differentiated	16 (19.8)	32.56 ± 4.88			
**TNM STAGE**					
I/II	33 (40.7)	72.42 ± 6.86	0.001		
III/IV	48 (59.3)	41.40 ± 4.53			
**TUMOR SIZE**					
≤4 cm	48 (59.3)	63.26 ± 5.78	0.014		
>4 cm	33 (40.7)	41.36 ± 5.49			
**LYMPH NODE METASTASIS**					
Negative (N0)	49 (60.5)	66.52 ± 5.69	0.000	0.331 (0.181–0.606)	0.000
Positive (N1–3)	32 (39.5)	35.44 ± 4.54			
**TOBACCO SMOKING**					
Non-smoker	29 (35.8)	49.55 ± 5.83	0.892		
Smoker	52 (64.2)	54.14 ± 5.35			
**ALCOHOL CONSUMPTION**					
Non-drinker	38 (46.9)	47.34 ± 5.07	0.625		
Drinker	43 (53.1)	56.74 ± 5.84			
**p16 STATUS**					
Negative	26 (32.1)	67.65 ± 7.60	0.070		
Positive	55 (67.9)	47.06 ± 4.45			
**PD-L1**					
Low	26 (32.1)	63.56 ± 5.67	0.007	0.289 (0.132–0.634)	0.002
High	55 (67.9)	46.75 ± 4.90			
**PD-1 ON LYMPHOCYTES**					
Negative	39 (48.1)	46.81 ± 4.92	0.585		
Positive	42 (51.9)	55.74 ± 5.80			
**FOXP3** **+** **Treg COUNT**					
Low	41	66.76 ± 5.82	0.009	0.412 (0.224–0.759)	0.004
High	40	40.29 ± 4.63			

**Table 6:** Univariate and multivariate analysis of clinicopathological factors in regard to DFS in patients with HNSCC.

**Characteristics**	**Cases, *n* (%)**	**Univariate**		**Multivariate**	
		**Survival time (month)**	***P*-value**	**HR (95% CI)**	***P*-value**
**GENDER**					
Male	65 (80.2)	40.00 ± 4.18	0.242		
Female	16 (19.8)	51.19 ± 7.92			
**AGE (YEARS)**					
< 60	49 (60.5)	42.13 ± 4.88	0.963		
≥60	32 (39.5)	41.73 ± 5.70			
**LOCALIZATION**					
Oropharynx	17 (21.0)	36.98 ± 6.66	0.751		
Non-oropharynx	64 (79.0)	41.85 ± 4.18			
**HISTOLOGY**					
Well differentiated	18 (22.2)	62.32 ± 7.91	0.024		
Moderately differentiated	47 (58.0)	37.86 ± 4.57			
Poorly differentiated	16 (19.8)	27.38 ± 5.69			
**TNM STAGE**					
I/II	33 (40.7)	57.60 ± 5.72	0.001	0.365 (0.191–0.697)	0.002
III/IV	48 (59.3)	31.78 ± 4.43			
**TUMOR SIZE**					
≤4 cm	48 (59.3)	50.37 ± 4.91	0.010		
>4 cm	33 (40.7)	30.71 ± 5.11			
**LYMPH NODE METASTASIS**					
Negative (N0)	49 (60.5)	51.50 ± 4.87	0.002		
Positive (N1–3)	32 (39.5)	27.84 ± 4.91			
**TOBACCO SMOKING**					
Non-smoker	29 (35.8)	44.22 ± 6.32	0.668		
Smoker	52 (64.2)	41.17 ± 4.69			
**ALCOHOL CONSUMPTION**					
Non-drinker	38 (46.9)	42.30 ± 5.52	0.983		
Drinker	43 (53.1)	42.19 ± 5.18			
**p16 STATUS**					
Negative	26 (32.1)	52.27 ± 6.90	0.096		
Positive	55 (67.9)	37.18 ± 4.23			
**PD-L1**					
Low	26 (32.1)	60.68 ± 6.12	0.002	0.322 (0.154–0.673)	0.003
High	55 (67.9)	33.93 ± 4.28			
**PD-1 ON LYMPHOCYTES**					
Negative	39 (48.1)	38.81 ± 5.44	0.476		
Positive	42 (51.9)	44.77 ± 5.05			
**FOXP3** **+** **Treg COUNT**					
Low	41	50.34 ± 5.39	0.038	0.479 (0.269–0.854)	0.013
High	40	33.74 ± 4.91			

## Discussion

Traditional therapeutic methods have unsatisfactory curative effects on HNSCC. Despite improvement in operation techniques and targeted therapy for immune checkpoints, the prognosis of head and neck cancer patients remains poor. Therefore, there is an urgent need to explore the key mediators and the underlying molecular mechanisms associated with tumor growth, recurrence and metastasis after curative treatment like surgical resection. Recently, accumulated research suggests that inflammatory mediators may play a role either in tumor establishment or in progression. In other words, the tumor microenvironment can be described by the chronic inflammation process.

HMGB1 and HMGN1 are recently identified alarmins which are a subset of damage-associated molecular patterns capable of inducing both the recruitment and activation of dendritic cells (DCs) (8, 9). Then DCs can promote specific immune responses against many tumor-associated antigens. Particularly, HMGN1 has been shown to be critical for the generation of anti-tumor immunity against mouse thymoma and melanoma (10). In our study, we demonstrated that cytoplasmic expression of HMGB1 in tumor tissue was associated with CD3+ T lymphocyte count but were not correlated with the count of FOXP3+ T cells, while HMGN1 in cytoplasm was correlated with CD3+ T cell count, CD8+ T cell count and FOXP3+ regulatory T cell count, suggesting that the secretion of these two HMG alarmins in tumor tissue may contribute to the influx of TILs.

Although our results demonstrated that secretion of HMG alarmins by tumor cells could recruit CD3+ and CD8+ T cells into tumor microenvironment, further analysis showed that the count of CD3+ and CD8+ T cells were not directly associated with postoperative survival. Meanwhile, the prognostic significance of HMG alarmins expression in HNSCC tissues has not yet been proven. However, our present work indicated that tumors with high FOXP3+ Tregs infiltration tended to have a poorer prognosis. This is in accordance with the previous studies which indicated that regulatory T cells played direct roles in promoting immune evasion and were correlated with poor prognosis in cancer (11). The infiltrated Treg cells in tumors, as one of the major immunosuppressive cellular population in the tumor microenvironment, accelerate the progression of malignant tumors by promoting tumor growth, enhancing tumor-associated angiogenesis, inhibiting adaptive immunity, and helping tumor cells invade surrounding normal tissues and metastasize to distant organs.

Recently, accumulating studies have verified the correlation between TILs and survival in several kinds of tumors. However, the prognostic role of TILs is still controversial. Some TIL subsets, especially CD8+ TILs, could be regarded as valuable prognostic factors in HNSCC (12). In our present study, though TILs infiltration is associated with HMG proteins, no statistical correlation with postoperative survival was observed. It may be ascribed to the expression of immune inhibitory molecules. Then, we wondered how the relationship of each molecule to survival related to tumor lymphocyte infiltration. The checkpoints of PD-1 and PD-L1 have crucial roles in the formation of “immune privilege” regions, tumor development, and immune evasion. PD-L1 is expressed in numerous tumors, including HNSCC. Binding of PD-L1 to its receptor PD-1, on activated T cells, suppresses T-cell responses (13). Our present work showed that high tumor expression of PD-L1 was an independent predictor of poor HNSCC-specific survival. Similar to our results, high expression of PD-L1 was found to be associated with poor survival in melanoma (14), non-small cell lung cancer (15), colorectal (16), and renal cell cancer (17). On the other hand, other studies in melanoma (18), gastrointestinal-stromal tumors (19), and colorectal cancer (20) have shown the opposite. Lately, a meta-analysis suggested the correlation between PD-L1 within tumors and the prognosis may vary among different tumor types and by the type of tissue sampling used for assessment. Therefore, the expression of PD-L1 on tumor cells is well-known but in order to assess its function in the (anti-) tumor immune response and its association with PD-1 expression on tumor infiltrating immune cells, further investigation is needed.

As cancer molecular biology and immunology developed rapidly, targeted therapy for PD-1/PD-L1 axis has shown enormous development prospects for HNSCC treatment. In 2016, the results of two milestone trials ]KEYNOTE-012 (21, 22) and CheckMate 141 (23)] promoted the antagonists of PD-1 / PD-L1 axis such as pembrolizumab (MK-3475) and nivolumab (BMS-936558) which become the earliest targeted therapeutic drugs for HNSCC approved by the US Food and Drug Administration (FDA). At present, the two drugs have already been the fourth standard therapeutic method after surgery, radiotherapy, and chemotherapy for HNSCC.

In the current study, statistical analysis showed that the number of CD3+ T cells in HMGB1 cytoplasmic expression group was more than that in group without HMGB1 cytoplasmic expression, while both CD3+ and CD8+ T cell counts in HMGN1 cytoplasmic expression group were more than that in group without HMGN1 expression. Although HMGB1 and HMGN1 function both as chromatin binding proteins and as alarmins, they are otherwise completely distinct proteins and have some distinct roles. When released by tumor cells, extracellular HMGN1 function as a Th1-polarizing alarmin that attracts CD8+ CTLs and promotes the induction of adaptive antitumor immune responses. However, the up-regulated expression of PD-L1 on tumor cells could inhibit the antitumor activity of CD8+ CTLs. HNSCC patients with cytoplasmic expression of HMGN1 and high tumoral PD-L1 expression, which suggests adaptive immune resistance, may benefit from PD-L1 or PD-1 blockade. But our results showed that there was no significant difference in the number of CD8+ CTLs between the two groups with or without HMGB1 cytoplasmic expression in tumor tissues. Therefore, in our opinion, compared to patients with HMGB1 cytoplasmic expression, HNSCC patients with both HMGN1 cytoplasmic expression and high expression of PD-L1 would be expected to get more benefit from anti-PD-L1/PD-1 therapy. In our present work, we found that 31 out of 81 patients had positive cytoplasmic expression of HMGN1 in tumor tissue. Among these 31 patients, 20 patients' tumor tissue expressed PD-1 on lymphocytes, and 24 patients' tumor tissue highly expressed PD-L1. Taken together, there were totally 16 patients whose tumor tissues were harboring HMGN1 cytoplasmic expressed and PD-L1 positive tumor cells, as well as PD-1 positive tumor infiltrating lymphocytes (TILs). We assumed that HNSCC patients with this type of molecular feature may benefit the most from PD-1/PD-L1 blockade.

In our opinion, the present work has several strong characteristics. We have examined the expression of HMG proteins and immune inhibitory molecule PD-L1 in tumor, as well as surrounding TILs. Our study is first to examine the co-expression of HMG proteins and PD-1/PD-L1 in HNSCC. There are of course also limitations in our study. Firstly, we investigated the expression of HMG proteins, TILs infiltration and PD-1/PD-L1 status only in patients who have a chance to undergo surgery, the data were not available for patients with advanced HNSCC and further study is needed. Secondly, the consensus to determine positive PD-1/PD-L1 marker threshold on biopsy tissue specimens by IHC is still a challenge. Substitutive tests such as RNA-based assays can be used to demonstrate the effects of PD-1/PD-L1 status in prognostic significance of HNSCC. Further studies are required to confirm the importance of HMG alarmins in tumor microenvironment.

In conclusion, our study found that the release of high-mobility group alarmins in tumor tissues of HNSCC patients was correlated with the count of TILs, in other words, it might involve in cellular immunity. However, further analysis suggested that PD-1/PD-L1 axis, rather than HMG alarmins or CD8+ tumor-infiltrating lymphocytes, associated with survival. Our results may pave a way for evolution of immunotherapy of head and neck cancer.

## Ethics Statement

The study was based on the recommendations from biomedical research guidelines involving human participants designed by the National Health and Family Planning Commission of China. The study is approved by the Ethical Committee of TMUCIH, and we obtained written informed consent from each subject on the basis of the Declaration of Helsinki.

## Author Contributions

XR, FW, and FY designed the study protocol. FY, ZZ, and JL performed the experiments. FY, YZ, and FW analyzed the collected data. FY wrote the manuscript. XR and FW revised the manuscript.

### Conflict of Interest Statement

The authors declare that the research was conducted in the absence of any commercial or financial relationships that could be construed as a potential conflict of interest.
